# Quality of uncomplicated malaria case management in Ghana among insured and uninsured patients

**DOI:** 10.1186/s12939-014-0063-9

**Published:** 2014-07-24

**Authors:** Ama P Fenny, Kristian S Hansen, Ulrika Enemark, Felix A Asante

**Affiliations:** 1 Economics Division, Institute of Statistical, Social and Economic Research (ISSER), University of Ghana, Legon LG74, Accra, Ghana; 2 Department of Public Health, Aarhus University, Vennelyst Boulevard 6, Århus C, 8000, Denmark; 3 Department of Global Health and Development, Faculty of Public Health and Policy, London School of Hygiene and Tropical Medicine, 15-17 Tavistock Place, London, WC1H 9SH, UK

**Keywords:** Quality of care, Uncomplicated malaria, Patient exit survey, Malaria treatment protocol, Ghana

## Abstract

**Introduction:**

The National Health Insurance Act, 2003 (Act 650) established the National Health Insurance Scheme (NHIS) in Ghana with the aim of increasing access to health care and improving the quality of basic health care services for all citizens. The main objective is to assess the effect of health insurance on the quality of case management for patients with uncomplicated malaria, ascertaining any significant differences in treatment between insured and non-insured patients.

**Method:**

A structured questionnaire was used to collect data from 523 respondents diagnosed with malaria and prescribed malaria drugs from public and private health facilities in 3 districts across Ghana’s three ecological zones. Collected information included initial examinations performed on patients (temperature, weight, age, blood pressure and pulse); observations of malaria symptoms by trained staff, laboratory tests conducted and type of drugs prescribed. Insurance status of patients, age, gender, education level and occupation were asked in the interviews.

**Results:**

Of the 523 patients interviewed, only 40 (8%) were uninsured. Routine recording of the patients’ age, weight, and temperature was high in all the facilities. In general, assessments needed to identify suspected malaria were low in all the facilities with hot body/fever and headache ranking the highest and convulsion ranking the lowest. Parasitological assessments in all the facilities were also very low. All patients interviewed were prescribed ACTs which is in adherence to the drug of choice for malaria treatment in Ghana. However, there were no significant differences in the quality of malaria treatment given to the uninsured and insured patients.

**Conclusion:**

Adherence to the standard protocol of malaria treatment is low. This is especially the case for parasitological confirmation of all suspected malaria patients before treatment with an antimalarial as currently recommended for the effective management of malaria in the country. The results show that about 16 percent of total sample were parasitologically tested. Effective management of the disease demands proper diagnosis and treatment and therefore facilities need to be adequately supplied with RDTs or be equipped with well functioning laboratories to provide adequate testing.

## Introduction

Malaria remains a leading cause of morbidity and mortality in sub-Saharan Africa. Malaria remains a leading cause of childhood illness and death in sub-Saharan Africa with an under five annual mortality of approximately a million [1]. It is the most significant public health problem in Ghana where it accounts for 38 percent of all outpatient illnesses, 35 percent of all admissions, and 34 percent of all deaths in children under five years [2]. Effective case management of uncomplicated malaria is a major strategy for malaria control [1]. This requires appropriate clinical assessment, laboratory proof of the disease either by microscopy or rapid diagnostic test (RDT) prior to treatment with an effective antimalarial [1]. Moreover, the Abuja declaration of May 2006, aims at achieving and sustaining universal access to appropriate interventions for all populations at risk of malaria. As a result, the goal of malaria control in Ghana is to reduce morbidity and mortality by 75 percent by 2015 [3].

Unfortunately, health services from both public and private providers are of questionable quality, with long waiting times, inaccurate diagnosis, inappropriate prescription and advice and frequent drug stock-outs. The use of presumptive malaria diagnosis without laboratory support is a common diagnostic procedure for malaria in Ghana and this predisposes patients to poor quality of malaria diagnosis and treatment [4]. A number of studies have shown that a large proportion of suspected malaria cases in Africa are given an antimalarial following presumptive diagnosis based on clinical symptoms only and without a parasitological test. This results in massive over-diagnosis of malaria, waste of antimalarial drugs and delay in appropriate treatments which in some cases have serious consequences [5]-[8].

The National Health Insurance Act, 2003 (Act 650) established the National Health Insurance Scheme (NHIS) with the aim of increasing access to health care and improving the quality of basic health care services for all citizens, especially the poor and vulnerable. The NHIS represents a major development in health system financing in Ghana. Since its introduction in 2005, levels of utilisation of health services has increased, as had been anticipated with the implementation of the scheme [9],[10]. What was not anticipated clearly was the effect of this on infrastructure and staffing levels at health facilities. Some studies indicate that there has been limited improvement of facility infrastructure and staffing levels which has led to a strain on health workers [11]-[13]. It is unclear how the supply-side was strengthened to cope with these developments. There has been anecdotal information alleging that patients who are insured receive poor treatment at health facilities compared to non-insured. This could be due to the late reimbursement of claims by the National Health Insurance Authority (NHIA). Health facilities are more willing to serve the non-insured who make out-of pocket payments to the facilities for services rendered. The sustainability of the NHI scheme is in danger if members leave the scheme and further discourage others from enrolling.

To substantiate some of these anecdotal evidence, we needed to assess whether the insured and the non- insured are given the same standard of treatment when it comes to malaria care. First we assess the preparedness of facilities to manage the treatment of uncomplicated malaria. Secondly, we assess the adherence to malaria treatment guidelines noting any possible differences between the insured and uninsured groups. We hypothesise that both groups will receive the same treatment at the facility level. Finally we evaluate the association between patient satisfaction and quality of care received. Here, patient satisfaction relies on patients’ perception of satisfaction on what was observed and how they were treated by providers. Uncomplicated Malaria is defined as the presence of fever or a recent history of fever, in the absence of any signs of severe disease [14].

Effective case management of malaria in the private and public health facilities will ultimately reduce malaria-related morbidity and mortality in Ghana. The information generated by this study will help design policy measures to strengthen the treatment component of the malaria control strategy and initiate improvement programmes.

## Background

### Ghana’s health system delivery and structure

The Ministry of Health (MOH) supervises and controls the policy formulation, monitoring and evaluation of activities and programmes geared towards achieving the targets set out in the health sector. The Ghana Health Service is responsible for delivery of public health and clinical services. Health care delivery is provided by both the public and private (private-for-profit and private-not-for-profit) sectors, with the public sector organized according to national (teaching hospitals), regional (regional hospitals), district (district hospitals), sub-district (health centres) and Community-based Health Planning and Services (CHPS). At the sub-district level, health centres are the highest health facilities and first line of referral to the formal health services from the community clinic.

Beyond this setup, is an informal health care sector consisting of traditional practitioners who may be formally or informally trained. They include herbalists applying preparations from plant materials, naturopaths and homeopaths and spiritualists. Licensed and unlicensed druggists and traditional birth attendants (TBA) can also be found in this sector. Prior to the introduction of the NHIS, the user-fee regime encouraged many Ghanaians to self-medicate, often relying on drug pedlars or self-prescription of medication [15]. Recent studies from Ghana and other developing countries where malaria is endemic show that drug shops are willing to sell cheap and less effective drugs; willing to sell less than the full course and nearly always rely on presumptive diagnosis [16]-[18].

### Overview of the NHIS

The National Health Insurance Act, 2003 (Act 650) established the National Health Insurance Scheme (NHIS) with the aim of increasing access to health care and improving the quality of basic health care services for all citizens, especially the poor and vulnerable. The law establishing the scheme allows for the concurrently operation of District-Wide (Public) Mutual Health Insurance schemes, Private Mutual Health Insurance schemes and Private Commercial Health Insurance schemes. The scheme covers inpatient hospital care, outpatient care at primary and secondary levels, and emergency and transfer services. The benefit package covers about 95% of treatment cases in Ghana including malaria, cervical and breast cancer, surgical operations, physiotherapy, maternity care, dental care and eye care. The NHIS scheme has an exemption policy to ensure that the poor and other vulnerable groups have access to healthcare. The exempt groups are children under the age of 18 years, the elderly above the age of 70 and the indigent (poor). A new NHIA law, Act 852 was established in November 2012 to make some adjustments to the previous law (Act 650). For instance, mental patients who were not adequately covered under the scheme have been included. The benefit package has also been extended to include any relevant family planning package to be provided under the National Health Insurance Scheme. As the need to balance access with quality of care is important to the success of the scheme, the new law enjoins the NHIA to collaborate with the relevant agencies to ensure quality healthcare to members of the Scheme and carry out clinical audits.

In order to become a member of the NHIS one needs to be registered and issued with a Health Insurance Membership ID Card. One needs to pay the appropriate premium (except those belonging to the exempt group) to benefit from the National Health Insurance Scheme. Children under the age of 18 and the elderly above 70 years must pay the registration fee which covers the administrative expenses for the issuance of their membership cards. The indigent are however exempted from both the registration fees and premiums. Membership is subject to yearly renewal and members must present their valid health insurance cards at the health facilities to benefit from the scheme. All public health facilities are automatically accredited to the NHIS. However, private health facilities have to apply to the National Health Insurance Authority (NHIA) for accreditation to participate in the scheme. Some of the accreditation criteria entail the number of qualified health personnel, availability and quality of utilities such as regular supply of water and electricity. Patients with valid NHIS cards may choose to access care either from public or private accredited health providers in the district.

### Defining and measuring quality of care

The multi-faceted nature of healthcare and numerous stakeholders make quality of care a very difficult concept to define and measure [19]-[24]. To find a definition to capture all differing perceptions of what encompasses quality of care led the Institute of Medicine to define quality of care as “the degree to which health services for populations increase the likelihood of desired health outcomes and are consistent with current professional knowledge” [25]. In principle, quality is assured if patients can get the services they need and if the services provided are beneficial to them.

A number of frameworks have been developed in the past for quality of care assessment and many more have been formulated as variations of the former ones. They include the World Health Organisation (WHO) quality of care framework, the Bamako Initiative and more disaggregated approaches [19],[26]-[29]. The disaggregated frameworks have gained popularity because they try and capture the complexity and multi-dimensionality of quality of care [28]. This study adopts the Donabedian Model which has been widely used in the healthcare quality field and has been applied across a spectrum of medical specialties and illness diagnoses [19],[30].

The framework divides factors impacting quality into structures, processes and outcomes, connected by unidirectional arrows in that order. Structure includes all the factors that affect the context for health care delivery. The physical facility, equipment, human resources, as well as organisational characteristics such as supervision are some of the areas classified under structure. Structure is often easier to observe and measure. Process refers to the interaction between providers and their patients and relates to how provider tasks and clinical processes are both organized and performed. It also includes providers’ ability to communicate and build trust with patients [29]. Donabedian defines this as interpersonal care and it includes “the management of the social and psychological interaction between client and practitioner” [19]. Outcomes include the effects of healthcare on patients or populations, changes to health status, health behaviour, patient satisfaction and health-related quality of life [30]. However, accurately measuring outcomes that can be attributed exclusively to healthcare is very difficult [19].

Donabedian’s model is however criticised for its sequential and linear progression from structure to process and outcome. The model suggests a directional link from the structure to processes of care and finally to patient outcomes [31]. Some suggest that there are other important factors such as patient characteristics and environmental factors that need to be incorporated for a more complete evaluation of quality care [31]-[33]. The model however, allows researchers to draw conceptual models that are suitable to their own health systems incorporating in them all the aspects that are needed to holistically evaluate their health interventions [34]. On the other hand, examining the process of care itself rather than its outcomes also provides good information on how medical care is being applied. For instance, the examination of processes of care such as clinical history, physical examination and diagnostic tests; technical ability to perform diagnostic procedures, continuity of care and acceptability of care to the patient provide relevant information that could guide the process of improving outcomes.

### Conceptual framework

**Figure 1:**
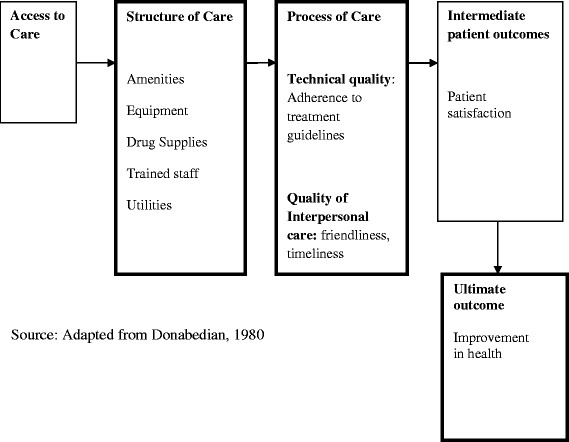
Donabedian’s structure-process-outcome paradigm.

Based on the Donabedian framework we construct a model to demonstrate the various links from structure to outcome (Figure 1). Structure of care includes amenities, equipment, drug supplies, utilities (water and power supply) and trained personnel. We define the process of care in terms of technical quality (adherence to clinical guidelines) and interpersonal aspects of care (attitude of staff towards clients). Measurement of process is often preferred because process is under relatively greater control of providers, needs a shorter time frame for results and can directly inform improvement [35]. Finally, we define patient satisfaction as an intermediate outcome which should lead to the ultimate outcome of health improvement.

### Malaria treatment protocol in Ghana

**Figure 2:**
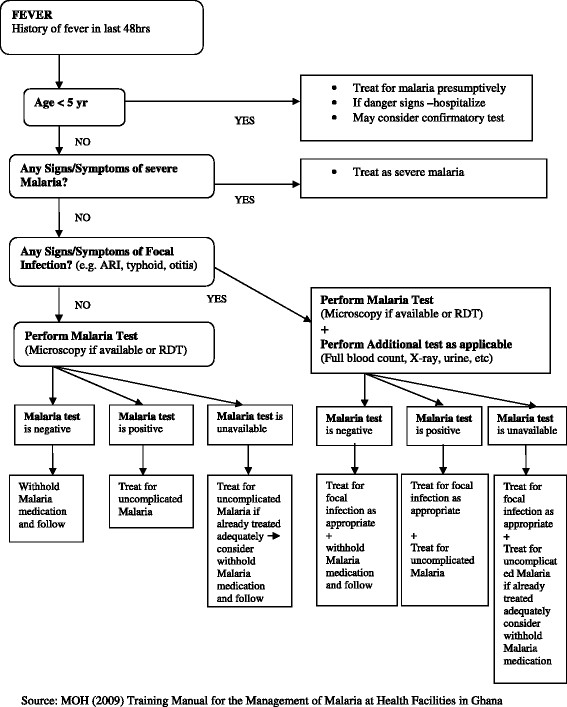
Flow chart for the diagnosis and treatment of uncomplicated malaria.

As part of process quality, the study focuses on the adherence to malaria treatment guidelines for uncomplicated malaria in Ghana. The standard for assessing the quality of malaria testing, diagnosis, and treatment in Ghana is presented in Figure 2. The flow chart presents all the different scenarios and steps to be taken in the treatment process. Uncomplicated malaria is mainly clinically diagnosed based on fever as case definition. In health facilities, the current approach is to confirm the clinical diagnosis with confirmation by parasitological test; either Rapid Diagnostic Test (RDT) or microscopy [14]. Currently, the use of RDTs is recommended for the diagnosis of malaria in lower level peripheral facilities (CHPS), but not in health centres and hospitals where microscopy is recommended. Microscopic testing should be the standard at health facilities, including district hospitals and higher level facilities. Laboratory services exist at most levels of health facility, except smaller health centres and therefore we expect facilities to be equipped to carry out the necessary diagnostic tests.

As noted in the standard protocol, in the initial assessment, the patient suffering from uncomplicated malaria commonly complains of: fever or a history of fever within the preceding 2–3 days, chills (feeling unusually cold), rigors (shivering) and headache. Other clinical features may include generalised body and joint pain, nausea and or vomiting, loss of appetite, sweating, abdominal pain (especially in children), bitterness in the mouth, irritability and refusal to feed (in infants). These features may occur one at a time or occur in combination. As part of the protocol a complete history is required from each patient and any presenting symptoms are noted. In patients with a suspected case of uncomplicated malaria, a parasitological confirmation is recommended, wherever possible, before giving antimalarial treatment (Figure 2).

The WHO Guidelines recommend a parasitological confirmation of diagnosis in all patients suspected of having malaria before treating [1]. The standard protocol for Ghana currently specifies that under-5 s with a febrile illness should be treated for malaria without testing (Figure 2). Other issues clarified include how patients should be managed if malaria testing is not available. However, in line with WHO guidelines the protocol highlights the importance of performing malaria tests and other tests where necessary before malaria medication is supplied to patients with the disease. Artesunate-Amodiaquine Combination is the combination drug of choice for the treatment of uncomplicated malaria as a first line treatment. The second line treatment is the recommended strengths and dosage forms of Artemether–Lumefantrine or Dihydroartemisinin Piperaquine [14].

### Study objectives

Based on the Donabedian framework, there are three main objectives in this study. The first is to assess the structural quality by considering the preparedness of facilities to manage the treatment of uncomplicated malaria. Process quality is assessed in two ways; technical quality where the adherence to malaria treatment guidelines is assessed and interpersonal quality which considers the attitude of personnel towards patients. The outcome assesses patient satisfaction with the overall quality of care received at the health facility.

## Methods

### Study design

The 10 administrative regions in Ghana are subdivided into 170 districts which cut across 3 agro-ecological zones in Ghana namely coastal, forest and savannah. For this study a district was selected in each zone making a total of 3 districts surveyed. The study was a cross-sectional survey consisting of provider interviews at selected facilities in the sample, an audit of staffing, equipment and procedures routinely conducted in antimalarial treatment prescribed to fever cases as well as exit interviews with clients through structured questionnaires.

Malaria is hyperendemic in all parts of the country, with all the 22.4 million population at risk. Transmission occurs all year round with slight seasonal variations during the rainy season from April to July [14]. The main parasite species causing malaria in Ghana are Plasmodium falciparum (80-90%), Plasmodium malariae (20- 36%), and Plasmodium ovale (0.15%). Mixed infections of P. falciparum and P. malariae are not uncommon [14].

### Provider interview

An initial mapping of the various types of health facilities provided in the district was conducted. The sample size was determined by considerations of the range of providers and feasibility. The sampling frame included the major types of private and public providers in each district. Public providers were district hospitals; health centres (HCs) and CHPS. All private providers in this study are accredited by the National Health Insurance Authority (NHIA) meaning that members with valid NHIS cards are able to benefit from approved services with no charge at the point use. The study sites for each district selected included the district hospital, 3 randomly selected health centres and 3 randomly selected community health posts (CHPS). The survey took place between January and March, 2011.

A structured questionnaire was administered to the heads or owners of selected public and private providers. The quality of antimalarial treatment prescribed to fever cases elicited from the various providers included service availability, human resource availability, and adherence to treatment standards and protocols (method of diagnosis of malaria; laboratory capacity and provision of ACTs and other anti-malarial drugs). This was based on the premise that appropriate treatment consists of proper diagnosis and treatment with nationally recommended drugs based on the guidelines. Other variables that were explored included assets (electricity, water, equipment) of the facilities and the general cleanliness of the environment.

### Exit interviews

#### Sampling for Exit interviews

The sample size for each district was 200 patients expected to be distributed equally between the insured and uninsured. Patients who reported being diagnosed with uncomplicated malaria and prescribed antimalarials were interviewed. The number of patients to be interviewed per selected health facility was obtained based on the daily average number of malaria cases presented in the health facilities. A random systematic sampling approach was used to select patient for interviews over the study period in each district. To start, the first patient was randomly chosen (e.g. the fourth patient who met with the criteria for selection). Subsequently, every second patient following the initially selected patient was sampled. Approximately 3–10 patients were interviewed a day depending on which type of facility (district hospital, health centre or CHPS) was surveyed. Data was collected on initial examinations performed (temperature, weight, age, blood pressure and pulse); observations of malaria symptoms by trained staff, laboratory tests conducted and types of drug prescribed. Insurance status of patients (whether patient was a valid cardholder), age, gender, education level and occupation were asked in the interviews.

### Ethical approval and consent

Ethical clearance was given by Institutional Review Board (IRB), of the Noguchi Memorial Institute for Medical Research (NMIMR). Informed consent was obtained from the respondent who read and agreed to be interviewed having accepted the conditions.

### Data analysis

Tabulations were used to examine variables for stated quality elicited from the various providers in the different areas. Tabulations using Chi-square test to examine adherence to treatment standards and protocols and differences between the insured and uninsured patients. This was based on the premise that appropriate treatment consists of proper diagnosis and treatment. These variables were the clients’ responses to whether specific processes of care were undertaken by trained personnel. Tabulations were also used to examine patient satisfaction with quality of care at the health facilities.

## Results

### Patient exit survey - demographic and background statistics

**Figure 3:**
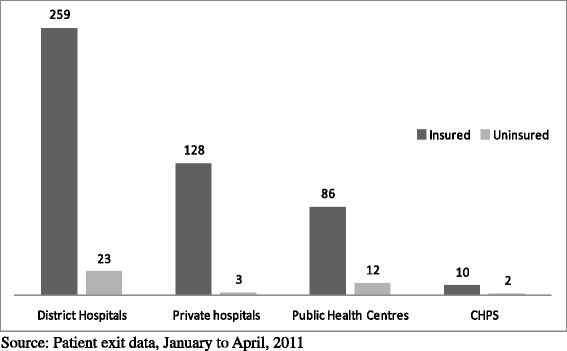
Distribution of outpatients interviewed by type of health care providers and insurance status.

Table 1 presents detailed findings of the demographic data and background characteristics. Our sample consisted of a total of 523 patients who were given treatment for malaria at the various facilities in all 3 districts. Out of the total sample 92 percent were insured and 8 percent non-insured; 64 percent males and 36 percent females. Patients under 5 years old accounted for 33 percent of the total sample with those above 70 years accounting for 4 percent. District hospitals accounted for the highest number followed by private hospitals and then health centres followed by the CHPS because of the sampling procedure. Out of the 40 uninsured patients only 3 sought care at private hospitals (Figure 3).

**Table 1:** Demographic and background statistics

**Variables**	**Frequency**	**%**
**Sex**		
Female	186	35.56
Male	337	64.44
**Age**		
<5	171	32.70
5-17 years	96	18.36
18-60 years	235	44.93
≥70 years	21	4.02
**Education**		
No education	132	39.76
Some primary	104	31.33
JSS/Middle	85	25.60
Secondary or higher	11	3.31
**Facility type**		
CHPS	12	2.29
Public health centres	98	18.73
Private hospitals	131	25.05
District hospitals	282	53.92
**Insurance status**		
Insured	483	92.35
Uninsured	40	7.65
**Nearest facility to patient**	319	60.99
Yes		
No	204	39.01
**N**	**523**	

Source: Patient exit data, January to April, 2011.

### Structure quality of care

#### General characteristics of the providers

Four main providers were assessed for the study: district hospitals, private hospitals, health centres and CHPS. All the respondents of the survey were heads of the facilities visited. All the facilities had trained personnel who were appropriate for each type of facility. All the health centres and CHPS were manned by trained medical assistants or midwives. There were doctors available in all the hospitals visited.

### Availability of functional equipment

All the facilities had functioning thermometers, weighing scales and cold boxes/fridges (Table 2). Essential malaria drugs including ACTs were available at the time of the survey with the exception of the CHPS compound where the facility was not equipped to handle severe malaria cases and therefore lacked quinine and intravenous (IV) fluid (Table 2).

**Table 2:** Availability of equipment, and medicines needed to manage malaria in outpatient health facilities

	**District Hospital**	**Private hospital**	**Public health centres**	**CHPS**
	**N = 3**	**N = 1**	**N = 7**	**N = 1**
**Characteristics**				
Thermometer	3	1	7	1
Functional scale for weighing	3	1	7	1
Cold box/fridge	3	1	7	1
**Laboratory capacity**				
Medical laboratory	3	1	4	0
Staff trained to perform microscopy	3	1	4	0
Functional microscope, according to the laboratory technician	3	1	4	0
Staff trained to perform RDTs	3	1	7	1
Malaria testing, by microscopy	3	1	4	0
Malaria testing, by both microscopy and RDT	2	1	2	1
**Medicines in stock**				
ACTs	3	1	6	1
Quinine	3	1	6	0
I.M. Artemeter	3	1	5	1
IV -Fluid	3	1	6	0
Artemisinin-based suppository	2	1	4	1

Source: Patient exit data January to April, 2011.

### Laboratory capacity

All the facilities had trained personnel on how to use RDTs if there were no laboratories available. Four out of the 7 public health centres (57%) had no medical laboratories and therefore referred severe malaria cases to the district hospital. Only 2 out of the 7 health centres had RDTs in stock and therefore could offer the RDT to patients (Table 2). The rest had run out of stock and were still waiting for supplies from the Central Medical Stores (CMS).

### Process quality of care

#### Quality of clinical assessment and use of diagnostic testing

The vital statistics are often taken by nurses or health care assistants prior to consultation with trained personnel (doctors, nurses, midwives or medical assistants depending on which type of facility is visited). Routinely, the client’s age, weight, temperature, pulse and blood pressure are recorded. The results show that in all the different types of facility a high proportion of clients had their temperatures taken and weight recorded (Table 3). The least recorded vital statistics was the pulse. There were significant differences between the insured and uninsured groups with the exception of taking of blood pressure and pulse.

**Table 3:** Vital statistics observed by different health care providers

**Vital statistics**	**Insured**	**Uninsured**
	**N = 483 (%)**	**N = 40 (%)**
**Temperature**	78**	97**
**Age**	49**	80**
**Weight**	95*	88*
**Blood pressure**	53	43
**Pulse**	27	38

*p-* Chi-square test.

**Significant at 1%.

*Significant at 5%.

Source: Patient exit data January to April, 2011.

The quality of clinical assessments was evaluated by measuring the proportion of patients for whom health workers had ascertained if a given sign or symptom was present. This could be elicited from the response given to a question asked or provided by the client spontaneously during consultation. In general, assessments needed to identify suspected malaria were low in all the facilities with convulsion ranking the lowest. There were no significant differences between the two groups for most of the symptoms checked except with loss of appetite and yellowish urine (Table 4).

**Table 4:** Malaria symptoms that the insured and uninsured patients were asked about by providers

**Malaria symptoms**	**Insured**	**Uninsured**
	**N = 483 (%)**	**N = 40 (%)**
**Hot body/fever**	82	88
**Weakness**	53	58
**Cough**	62	55
**Vomiting**	58	65
**Convulsion**	6	5
**Joint pains**	35	30
**Nausea**	21	23
**Chills and shivering**	41	48
**Yellowish eye ball**	40	35
**Catarrh**	21	25
**Diarrhoea**	50	50
**Headache**	58	58
**Loss of appetite**	36*	53*
**Bitter taste**	23	28
**Yellowish urine**	8*	20*

*p-* Chi-square test.

*Significant at 5%.

Source: Patient exit data January to April, 2011.

Parasitological assessment in all the facilities was very low with 4 percent of the insured and 10 percent of the uninsured tested using RDTs. For laboratory testing, 15 percent of the insured were tested for malaria compared to 25 percent of the uninsured (Table 5).

**Table 5:** Parasitological test performed

**Laboratory assessment (RDT or microscopy )**	**Insured**	**Uninsured**
	**N = 483 (%)**	**N = 40 (%)**
**Yes**	19	35

Source: Patient exit data January to April, 2011.

### Outcome quality of care

#### Patient satisfaction

In total, 93 percent were satisfied with overall care and 7% were not satisfied. Similar trend was reported in the insured and uninsured groups. Satisfaction levels were highest in the health centres where 98 percent of those attending the facilities were satisfied with overall care (Table 6). There were no clear differences between the two groups in relation to the quality of care variables with the exception of waiting time and satisfaction at the pharmacy/dispensary (Table 7). Proportionally, more of the uninsured patients were satisfied with the waiting time and the pharmacy/dispensary compared to the insured (73% vs. 62%). The perception of quality of care received at a facility influences decisions as to whether to return and whether to recommend the service to other potential users (family members or friends). From the patient exit survey, about 98 percent of the total number would return to the health facility and will also recommend the facility to friends and family. This was the same in both the uninsured and insured groups (Table 8).

**Table 6:** Number of patients (%) who were satisfied with overall care, patient exit survey

	**N**	**Satisfied (%)**	**Not satisfied (%)**
Total	523	93.3	6.7
**Insurance status**			
Insured	483	93.6	6.4
Uninsured	40	90	10
*p* = 0.384 χ^*2*^ = 0.759			
**Facility type**			
District Hospital	282	92.2	7.8
Private hospital/clinic	131	91.6	8.4
Health centres/CHPS	110	98.2	1.8
*p* = 0.069 χ^*2*^ = 5.350			

Source: Patient exit data January to April, 2011.

**Table 7:** Number of patients (%) who were satisfied with care by quality of care dimensions and health insurance status

**Quality of care variables**	**N**	**Satisfied (%)**	**Not satisfied (%)**
**Waiting time**			
Insured	483	61.5	38.5
Uninsured	40	72.5	27.5
*p* = 0.167 χ^*2*^ = 1.907			
**Friendliness of staff**			
Insured	483	97.5	2.5
Uninsured	40	97.5	2.5
*p* = 0.849 χ^*2*^ = 0.327			
**Satisfaction at reception**			
Insured	483	55.8	44.2
Uninsured	40	57.5	42.5
*p* = 0.836 χ^*2*^ = 0.043			
**Satisfaction with consultation**			
Insured	483	97.9	2.1
Uninsured	40	97.5	2.5
*p* = 0.856 χ^*2*^ = 0.033			
**Satisfaction at pharmacy/dispensary**			
Insured	483	64.6	35.4
Uninsured	40	72.5	27.5
*p* = 0.313 χ^*2*^ = 1.017			

Source: Patient exit data January to April, 2011.

**Table 8:** Number of patients (%) who received all medicines, were asked for follow-up, will revisit facility and recommend facility to friends/relatives

	**N**	**Yes (%)**	**No (%)**
**Received all prescribed medicines**			
Insured	483	94.2	5.8
Uninsured	40	95	5
*p* = 0.835 χ^*2*^ = 0.043			
**Follow-up review requested**			
Insured	483	43.1	56.9
Uninsured	40	45	55
*p* = 0.812 χ^*2*^ = 0.056			
**Will revisit facility**			
Insured	483	97.5	2.5
Uninsured	40	87.5	12.5
*p* = 0.001 χ^*2*^ = 11.783			
**Will recommend facility to friends/relatives**			
Insured	483	97.7	2.3
Uninsured	40	87.5	12.5
*p* = 0.000 χ^*2*^ = 13.017			

Source: Patient exit data January to April, 2011.

## Discussion

This study sets out to ascertain the significant differences between the quality of structure, process and outcome of care for antimalarial treatment prescribed to fever cases among insured and uninsured patients compared to the standard protocol outlined in the guidelines for the treatment of malaria. A limitation of the present study is that the low number of uninsured in the exit interview did not allow for a more rigorous assessment of possible differences between the insured and uninsured. The GHS 2011 annual report shows that the proportion of OPD attendance by insured clients increased from 55.8 percent in 2010 to 82.1 percent in 2011 [2]. It was assumed at the start of the study that there may be almost equal numbers of the insured as uninsured. Notwithstanding this, the information shows what truly exists on the ground. The fact that majority of respondents were card holders confirms the results of previous studies [9],[10].

Going by Donabedian’s ‘structure-process-outcome’ model, we identify a number of shortfalls with case management of malaria among outpatients with uncomplicated malaria. First is the use of presumptive malaria diagnosis without laboratory support, which seems to be a common diagnostic procedure in both public and private facilities surveyed. Although majority observed vital statistics, very few of the symptoms of malaria were checked for. In addition, less than 15 percent of the patients in total were diagnosed and treated for uncomplicated malaria in the public and private health facilities based on test results from microscopy or RDTs. Presumptive treatment for malaria seems to be widely practised across all levels of healthcare provision with the exception of CHPS where RDTs were available and used on all occasions.

The standard protocol highlights the need for systematic laboratory testing but clearly this has not been adhered to in the health facilities. This phenomenon is not restricted to Ghana; other African countries where malaria is endemic report the very low rates of the use of malaria diagnostics. Dodoo et al. in a study from 2007 to show the mode of diagnosis and pattern of drug management in outpatients diagnosed with suspected uncomplicated malaria, indicate that only 3.2 percent of total diagnoses were parasitologically diagnosed [36]. Nationally, there has been reported improvement in the percentage of OPD malaria cases tested, from 14 percent in 2009 to 18.9 percent in 2011 [2]. However, the problem is even more entrenched in cases where providers have been known to have prescribed malaria drugs when microscopy cases have been shown to be negative [5],[6],[8],[37].

The study shows that although, all the structure elements are often available it doesn’t guarantee that the process elements will be followed through. For instance all the district hospitals and the private hospital in the sample had medical laboratories with functional microscopes and trained staff to perform malaria testing by microscopy yet very few of them parasitologically diagnose the malaria cases. There is clearly an adequate provision of essential diagnostic services (microscopy or RDTs) for malaria diagnosis in the health facilities but little use and this suggests very weak monitoring of the quality of malaria diagnostics.

On the outcomes, patient satisfaction ratings were high with many of them willing to revisit and recommend the facilities to friends and families. There was significantly no difference between the insured and uninsured patients. However, more of the insured patients were dissatisfied with the waiting time and the services offered at the pharmacies/dispensaries. We are limited in making any conclusive judgments because of the very small numbers of the uninsured. Yet the results of a similar study in two districts in the northern Ghana indicate that the long delay at pharmacies was one of the major complaints by insured patients [38].

## Conclusion

Compared to the national malaria protocol for the treatment of malaria and the current recommendation by the WHO on parasitological testing of all febrile cases, this study has revealed a clear short coming in the process care of the treatment of uncomplicated malaria in the selected facilities. The quality in terms of undertaking of routine microscopy was poor in all health facilities, regardless of their level. Some public health centres had no laboratories at all although this is vital to the performance of malaria diagnostic tests in the country. Treating all fevers presumptively as malaria can hide underlying fatal conditions. We recommend that these facilities are equipped with well functioning laboratories to provide adequate testing. It is also recommended to improve the capacity for RDT use in facilities where laboratories have not been provided with technical assistance provided.

The fact that very few uninsured patients presented with uncomplicated malaria cases limits our comparative analysis of antimalarial treatment prescribed to fever cases between the two groups. However, the very few numbers of uninsured raises red flags, as we have no a priori reason to believe that the uninsured suffer less from malaria than the insured. We are not sure where the uninsured are seeking care. Treatment interventions meant to reduce malaria mortality or morbidity is most often facility-based and may therefore be missed by the ill who seek care outside of the health facilities. Patient satisfaction levels were quite high whether by insurance or facility type but more information about quality differentials across providers is needed to identify the key areas for intervention. The study design was such that we may have missed a number of dissatisfied patients who no longer used the facilities and therefore the patient satisfaction ratings may have been slightly exaggerated. We recommend further studies to allow for more analysis on the quality of healthcare services for antimalarial treatment prescribed to fever cases.

## Competing interests

The authors declare that they have no competing interests.

## Authors’ contributions

APF was involved in the study design, training of research assistants, review of data collection tools, data collection, data entry, data analysis, drafting the manuscript, revising and writing of the final manuscript. KSH, UE and FAA contributed to the design, review of data collection tools, data analysis, critical review of the manuscript and revising the manuscript. All authors read and approved the final manuscript.
